# A method to quantify mechanobiologic forces during zebrafish cardiac development using 4-D light sheet imaging and computational modeling

**DOI:** 10.1371/journal.pcbi.1005828

**Published:** 2017-10-30

**Authors:** Vijay Vedula, Juhyun Lee, Hao Xu, C.-C. Jay Kuo, Tzung K. Hsiai, Alison L. Marsden

**Affiliations:** 1 Department of Pediatrics (Cardiology), Stanford University, Stanford, California, United States of America; 2 Department of Bioengineering, University of California, Los Angeles, Los Angeles, California, United States of America; 3 Department of Electrical Engineering, University of Southern California, Los Angeles, California, United States of America; 4 Department of Medicine, Division of Cardiology, University of California, Los Angeles, Los Angeles, California, United States of America; 5 Department of Bioengineering, Stanford University, Stanford, California, United States of America; 6 Institute for Computational and Mathematical Engineering (ICME), Stanford University, Stanford, California, United States of America; University of Pennsylvania, UNITED STATES

## Abstract

Blood flow and mechanical forces in the ventricle are implicated in cardiac development and trabeculation. However, the mechanisms of mechanotransduction remain elusive. This is due in part to the challenges associated with accurately quantifying mechanical forces in the developing heart. We present a novel computational framework to simulate cardiac hemodynamics in developing zebrafish embryos by coupling 4-D light sheet imaging with a stabilized finite element flow solver, and extract time-dependent mechanical stimuli data. We employ deformable image registration methods to segment the motion of the ventricle from high resolution 4-D light sheet image data. This results in a robust and efficient workflow, as segmentation need only be performed at one cardiac phase, while wall position in the other cardiac phases is found by image registration. Ventricular hemodynamics are then quantified by numerically solving the Navier-Stokes equations in the moving wall domain with our validated flow solver. We demonstrate the applicability of the workflow in wild type zebrafish and three treated fish types that disrupt trabeculation: (a) chemical treatment using AG1478, an *ErbB2* signaling inhibitor that inhibits proliferation and differentiation of cardiac trabeculation; (b) injection of *gata1a morpholino oligomer* (*gata1aMO*) suppressing hematopoiesis and resulting in attenuated trabeculation; (c) *weak-atrium*^*m*58^ mutant (*wea*) with inhibited atrial contraction leading to a highly undeveloped ventricle and poor cardiac function. Our simulations reveal elevated wall shear stress (WSS) in wild type and AG1478 compared to *gata1aMO* and *wea*. High oscillatory shear index (OSI) in the grooves between trabeculae, compared to lower values on the ridges, in the wild type suggest oscillatory forces as a possible regulatory mechanism of cardiac trabeculation development. The framework has broad applicability for future cardiac developmental studies focused on quantitatively investigating the role of hemodynamic forces and mechanotransduction during morphogenesis.

## Author summary

We present a novel computational workflow for quantifying hemodynamic forces in developing zebrafish embryos by coupling high resolution 4-D light sheet imaging with a moving domain blood flow solver. Our framework employs deformable image registration to extract the motion of the ventricle from high resolution image data. This produces a robust and efficient workflow, as segmentation is performed at only one cardiac phase, while the wall position in other cardiac phases is found from the displacement field obtained during image registration. This approach avoids a laborious process of manual segmentation in all cardiac phases, and minimizes spurious errors arising from manual processing. Our validated flow solver is optimized for cardiac hemodynamics with backflow stabilization, efficient data management and dynamic remeshing algorithms for moving domains. We demonstrate the utility of the framework in wild type zebrafish and three treated variants in which the formation of cardiac trabeculations is disrupted. In this study, we then quantify the relationship between oscillatory shear forces and the presence or absence of ventricular trabeculation during cardiac development. Our framework has broad applicability in cardiac developmental studies focused on quantitatively investigating the mechanobiology during morphogenesis.

## Introduction

Ventricular trabeculation is tightly regulated by both genetic programming and biomechanical forces such as hemodynamic pressure and shear stress. [1–8] Trabeculae formation leads to a complex network of endocardial protrusions (trabeculae) into the ventricle that form ridges and grooves. [9] During cardiac morphogenesis, the ventricular myocardium (heart tissue) differentiates into two layers, an outer compact zone and an inner trabeculated zone. Disruptions in any of the normal developmental processes can lead to either excess trabeculation, a congenital condition known as non-compaction cardiomyopathy, [7, 10–13] or a significant reduction in trabeculation that is usually associated with ventricular compact zone deficiencies such as hypoplastic left heart syndrome (HLHS). [1, 14] Both these conditions can lead to heart failure and high mortality during embryonic development. While the genetic mechanisms underlying cardiac morphogenesis have been extensively studied, the impact of biomechanical forces such as hemodynamic shear remains elusive, due in part to the significant challenges associated with quantifying hemodynamic forces in developing hearts. [3, 4, 7, 15]

Several studies have examined mechanotransduction during ventricular trabeculation using *in vitro* techniques such as particle image velocimetry (PIV). [4, 16, 17] Although non-invasive, these 2-D image-based techniques are limited by interpolation errors that arise when extracting the three-component (3C) velocity vector field as well as the lack of resolution of the near-wall velocity profile. [4, 17] These challenges compromise the accuracy of endocardial wall shear stress (WSS) measurements, which are of central importance to understanding shear-regulated mechanotransduction. Moreover, extracting hemodynamic pressure data, which is linked to cardiac valvulogenesis, [5, 18] from PIV measurements is non-trivial. Computational fluid dynamics (CFD) provides an attractive alternative for quantifying space-time resolved velocity and pressure fields in subject-specific geometries. CFD has been widely applied to simulate blood flow, to facilitate clinical decision-making, and to study the progression of cardiovascular disease. [19–26] CFD has also been applied to study developmental dynamics in chick embryos such as aortic arch morphogenesis, [27, 28] aortic valve and outflow tract morphogenesis, [29–31] and the onset of congenital heart disease such as HLHS. [14]

We have previously demonstrated, using moving domain CFD coupled with *in vivo* imaging of zebrafish embryos, a method to computationally quantify the spatio-temporal variation of endocardial WSS and pressure gradients across the atrio-ventricular canal in two dimensions. [32] We subsequently developed 4-D imaging (3-D in space + time) using light sheets with selective plane illumination microscopy (SPIM) coupled with a non-gated synchronization algorithm to elucidate hemodynamic regulation mechanisms of *Notch* signaling pathways during cardiac trabeculation in genetically manipulated zebrafish embryos. [33] While numerous image-based CFD modeling techniques have been developed for human hearts based on magnetic resonance imaging (MRI) or computed tomographic (CT) data, [34–36] there remains a need for efficient frameworks applicable to cardiac developmental studies using high resolution embryonic heart images.

We present a computational framework to quantify biomechanical forces, including endocardial WSS and oscillatory shear index (OSI) in zebrafish embryos with and without cardiac trabeculation. We also compare kinetic energy density and rate of viscous energy dissipation due to changes in ventricular trabeculation. Our framework employs robust and efficient image processing techniques based on the open-source SimVascular [37] software framework to build the anatomic model, and employs validated stabilized finite element methods for blood flow simulation in moving domains. [37–41] We apply this computational framework to quantify in detail the shear regulation of cardiac trabeculation during morphogenesis in zebrafish embryos in response to genetic and chemical treatments. In the following sections, we present the computational pipeline which proceeds from 4-D image data to computing ventricular hemodynamics in zebrafish embryos. We provide details on the genetic and chemical treatments of the wild type zebrafish to investigate the role of shear on cardiac trabeculation. Finally, we quantify differences in hemodynamic conditions between the wild type and treated variants over the course of cardiac development. In the present study, we limit our attention to velocity-derived quantities such as WSS, OSI, kinetic energy and dissipation although other mechanobiology regulators such as pressure gradients and wall strains are also likely factors affecting cardiac morphogenesis.

## Methods

### Computational workflow

**Fig 1:**
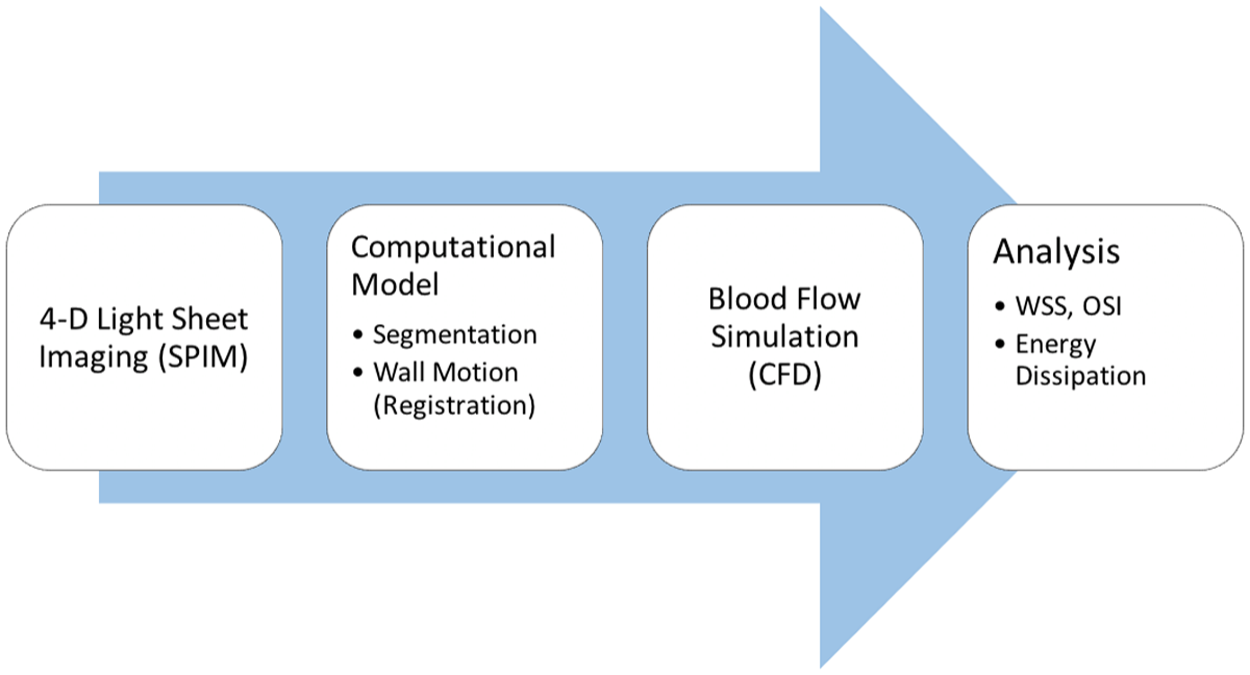
A schematic of the workflow for computing ventricular hemodynamics from 4-D image data in developing zebrafish embryos, proceeding from image acquisition to anatomic model generation and blood flow simulation and analysis.

We first present the computational workflow, which proceeds from 4-D light sheet images of zebrafish embryos to ventricular blood flow modeling using moving domain CFD (Fig 1).

#### 4-D light sheet SPIM imaging

The workflow begins with 4-D light sheet image acquisition using our in-house SPIM technique followed by a postprocessing synchronization step to visualize the dynamic cardiac structure at high spatial and temporal resolution. Details of the imaging system and synchronization algorithm are furnished in the Supplementary Material of Lee et al. [33]. Briefly, we scan approximately 70 sections from the anterior to the posterior ends of the zebrafish heart, where each section is captured with 500 snapshots (frames) at 10ms exposure time per frame via a sCMOS camera (Hamamatsu Photonics). The total acquisition duration is about 350s for each sample. The in-plane resolution of each frame is about 0.65*μm* × 0.65*μm* and the axial displacement along the z-direction is set to 2*μm*. [42] To synchronize with the cardiac cycle, we determined the cardiac periodicity on a frame-to-frame basis by comparing the pixel intensity from the smallest volume at end-systole to the largest volume at end-diastole. [33] Each reconstructed volumetric image post synchronization comprises 512 × 512 × 70 voxels and we acquire ∼100 volume images per cardiac cycle. The reconstructed 4-D image data sets were then processed in the Amira software (FEI, Inc.) for visualization. [33]

#### Computational model

The reconstructed 4-D zebrafish images are processed through a series of steps to extract the computational model of a beating ventricle for blood flow simulation.

**Fig 2:**
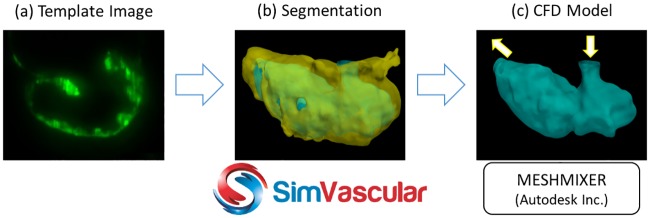
3-D image segmentation techniques in SimVascular [37] and surface tuning tools in Meshmixer (Autodesk Inc.) are leveraged to create anatomic models of the developing zebrafish endocardium for use in CFD simulations.

*Segmentation*: A template image is chosen as a starting point from the *M* frames of the 4-D zebrafish cardiac images (Fig 2a). Typically, the template is chosen during mid-diastole so as to be evenly positioned between minimum volume at end-systole and maximum volume at end-diastole. This template image is then segmented by thresholding based on the histogram of image intensities to create an isosurface of the ventricular myocardium. Since the choice of threshold value is user-dependent, we deform the initial threshold-based segmentation using level-set advection techniques to conform to the true edges of the image. We perform the 3-D level-set segmentation in the SimVascular open source image-based cardiovascular flow modeling software (Fig 2b). [37] For the final clean-up and to ensure a fluid-tight volume for blood flow simulations, we then import the level-set based segmentation from SimVascular as a triangulated surface into MeshMixer (Autodesk, Inc.). (Fig 2c) We extract the ventricular endocardium, filling any holes created during segmentation, and perform local smoothing and remeshing to eliminate sharp corners or intersecting surface triangles. Further, we slightly extrude the inlet and outlet orifices and cap them to prescribe inflow and outflow boundary conditions (Fig 2c).

*Registration, Motion Extraction*: Zebrafish embryos typically beat at high heart rates (∼120-150 beats per min) and undergo complex, large deformation endocardial motion, posing a significant challenge for motion extraction. One feasible approach is to repeat the segmentation for each image frame throughout the cardiac cycle. However, the triangulated surface mesh topology, including surface nodes and element connectivity, are not guaranteed to correlate from one frame to the next. Others have addressed this issue by performing a template-based mapping, either using a point-set registration technique or by applying large deformation mapping algorithms to extract the endocardial motion. [34, 43] Although these techniques have been successfully applied in human heart models based on MRI or CT data sets with relatively low temporal resolution (∼20 frames per cycle), they are inefficient for developmental studies with higher frame rates (∼100 frames per cycle). Moreover, manually segmenting all cardiac frames is a laborious process that substantially increases the workload, leading to segmentation errors and variability.

We circumvent this problem by leveraging image registration techniques hailing from the medical imaging and computer vision communities. We employ an intensity-based non-rigid deformable image registration method for extracting the motion of the ventricular endocardium from 4-D light sheet images by adapting the MATLAB-based open-source Medical Image Registration Toolbox (MIRT). [38] The toolkit has been validated for cardiac ultrasound images using sonomicrometry-based measurements of ventricular strains. [44] With this approach, we segment the endocardium at only one cardiac phase, and then morph the segmented surface using displacements computed from image registration. In this way, we not only reduce user effort by avoiding the need to segment all time frames, but also minimize segmentation errors and streamline the workflow.

We present a brief overview of the registration procedure here and guide the reader to [45] for a more detailed discussion of the algorithms and point-set mapping techniques. Given a source image *I*(*x*, *y*, *z*) and a target (or reference) image *J*(*x*′, *y*′, *z*′), our goal is to find an optimal transformation $T:\left(x,y,z\right)\mapsto \left(x^{\prime },y^{\prime },z^{\prime }\right)$ that maps a given anatomical point from the source image to the target image. We note that image registration represents a geometric transformation of the image and not an intensity transformation. There are three essential components required for performing image registration: (a) a similarity function **E**_**sim**_, (b) a transformation model T, and (c) a regularization function **E**_**reg**_. The similarity function **E**_**sim**_ defines the objective function to be minimized which aligns the two images, and the transformation model T defines the mapping of each individual point between the two sets of image coordinates. Regularization **E**_**reg**_ is essential to make the problem well-posed and provide control on the degree of deformation. Therefore, the objective function to be minimized is given by

$$\begin{array}{c} E_{\text{obj}}=E_{\text{sim}}(I(x),J(T(x)))+\lambda E_{\text{reg}}(T(x)), \end{array}$$ (1)

where λ is a regularization parameter that determines the trade-off between the accuracy of image alignment and the smoothness of the deformation field.

We use the sum of squared differences (SSD) similarity function, $E_{sim}^{SSD}=\frac{1}{N}\overset{N}{\underset{n=0}{\sum }}{\left[I\left(x_{n}\right)-J\left(T\left(x_{n}\right)\right)\right]}^{2}$, which is widely used in serial MR imaging, with the assumption that the images differ only by Gaussian noise. Although there are other complex similarity functions that take into account the imaging modality and precise noise representation, [44] our experience has shown that the SSD similarity measure produces reasonable results when applied to 4-D light sheet images. We use a free-form deformation (FFD) transformation model based on cubic B-splines, [46, 47] which deforms an object by manipulating a mesh of control points, interpolated using B-spline basis functions. These basis functions provide local support with low computational cost while producing a smooth and $C^{2}$ continuous transformation. The regularization employed in the current study is based on the squared norm of the gradient of the transformation, $E_{\text{reg}}=\frac{1}{2}{\left\|\nabla T\left(x\right)\right\|}^{2}$, which is associated with the elastic bending energy of the deformation. Finally, we use a gradient-descent method to minimize the cost function **E**_**obj**_ (Eq 1), implemented using a hierarchical multiresolution approach in which the resolution of the B-spline control point mesh is increased, along with the image resolution, from coarse to fine. [48] These multilevel or multigrid methods are widely used in image processing and fluid mechanics applications, and not only converge quickly to the optimal solution, but also capture local non-rigid deformations at a relatively low computational cost.

**Fig 3:**
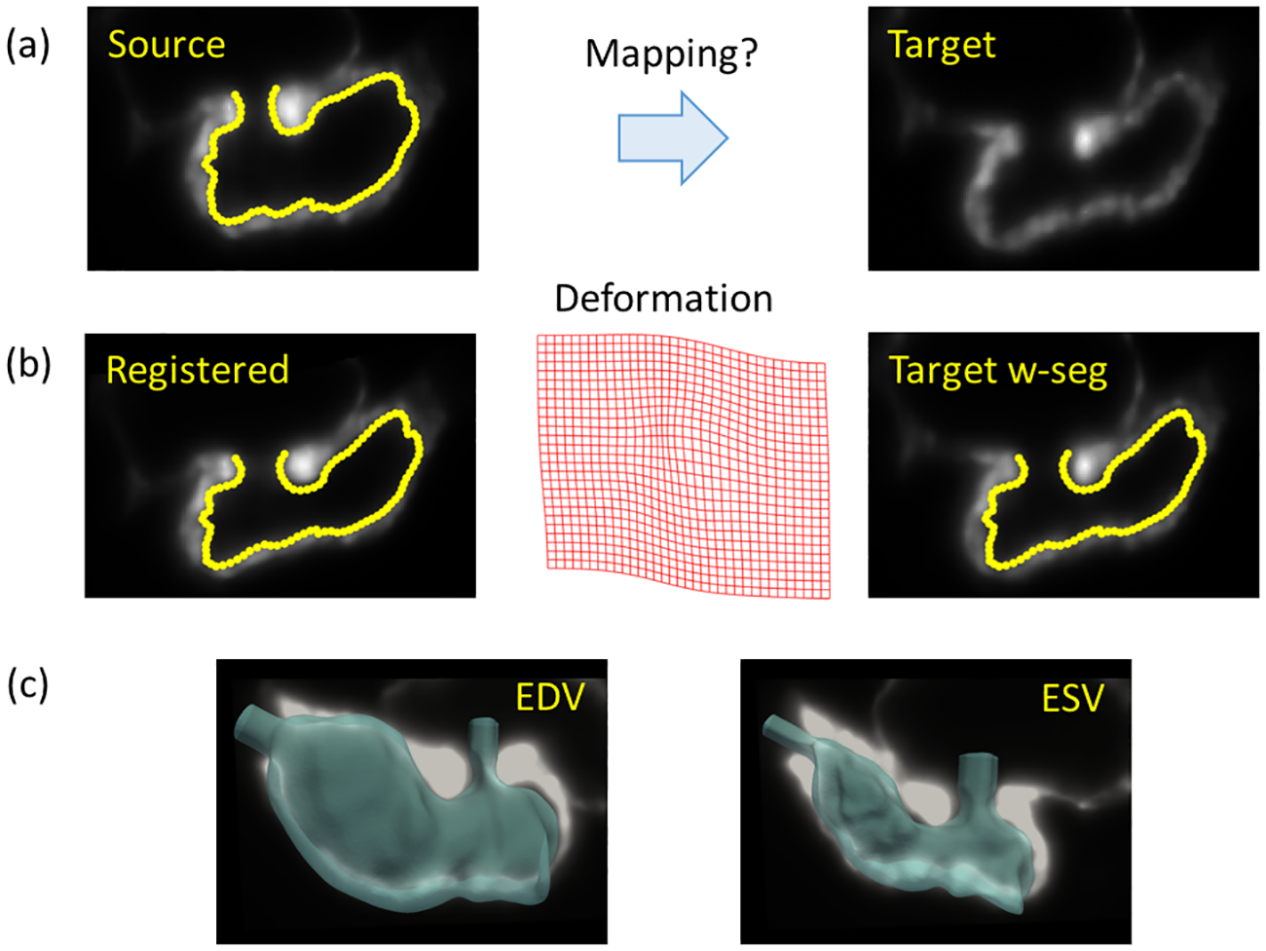
Intensity-based non-rigid deformable image registration methods are used to extract ventricular endocardial motion from 4-D light sheet image data. (a) We have chosen a source image with segmented endocardium at mid-diastole (left) and a target image at end-systole (right). (b) We perform registration using the MIRT framework to obtain the registered image (left), and compute the deformation field (middle) that is then used to morph the segmented endocardium. We note a reasonable agreement between the morphed endocardium boundary and the target image (right). (c) The registered 3D endocardial surfaces superposed on the corresponding background image are shown at end-diastolic (EDV, left) and end-systolic (ESV, right) phases of the cardiac cycle.

The registration process for the light sheet zebrafish images demonstrates good adherence to the image data (Fig 3, see S1 Movie in the Supplementary Material). We chose 4 sublevels to perform the B-spline based registration with a control point or knot spacing of 5 pixels and the regularization parameter (Eq 1), λ = 0.1. The tolerance for convergence during optimization was set to 10^−8^. Although the images were scanned at 512 × 512 pixels along each section, we cropped the image to the region of interest around the ventricle to reduce computational cost. Fig 3 shows source and target images which are far apart in the cardiac cycle, though in practice, we perform sequential registration on the 4-D light sheet images starting from mid-diastole and registering with each successive cardiac frame. We visually confirm reasonable agreement between the registered endocardium surface and the endocardial edges in the background image at end-diastolic (maximum ventricular volume, EDV) and end-systolic (minimum ventricular volume, ESV) phases of the cardiac cycle (Fig 3c).

To provide quantitative validation of the anatomic segmentation, we have manually segmented the ventricular wall boundary at 10 equidistant phases in the cardiac cycle and compared with the output of registration. While obtaining a local point-to-point agreement between these two surfaces is not practical, since the registration is based on a global minimization algorithm, we note a maximum of 10.9% (mean ∼8%) deviation in ventricular volume between the manually segmented and image registration-based ventricular surfaces. We believe that this difference is acceptable, in light of the other potential uncertainties in the simulation. Additionally, the method provides the benefit of substantially reducing the cost of model creation. For example, while it takes ∼2 hours to manually segmenting each endocardial surface, registering a pair of high resolution images takes only ∼2 minutes. Moreover, a point-set matching has to be performed on the manually segmented surfaces to apply Dirichlet boundary conditions, which can be very expensive. [34] We repeated the above process with the same registration parameters and tolerances for both wild type and treated zebrafish and obtained comparable results.

#### Blood flow modeling on moving domains

Blood flow in moving domains can be modeled using either interface-capturing immersed boundary (IB) methods [49–51], or interface-tracking arbitrary Lagrangian-Eulerian (ALE) methods. [40, 52] We simulate blood flow using the ALE approach, in which the interface is tracked along with a domain-conformal fluid mesh, represented by the moving endocardial wall and the discretized blood domain in the ventricular cavity, respectively. Here we present a brief account of the ALE methodology and refer the interested reader to [40, 41, 53] and the references contained therein for further details.

For an incompressible and Newtonian fluid, the weak formulation of the Navier-Stokes equations in ALE coordinates for moving domains is given as follows:

Find $\bar{v}\in S_{\bar{v}}$ and $p\in S_{p}$, such that for all test functions $\bar{w}\in V_{\bar{v}}$ and $q\in V_{p}$,

$$\begin{array}{c} B_{G}\left(\left\{\bar{w},q\right\};\left\{\bar{v},p\right\};\hat{v}\right)=F_{G}\left(\bar{w}\right)\;\;\text{where}, \end{array}$$ (2a)

$$\begin{array}{ll} B_{G}\left(\left\{\bar{w},q\right\};\left\{\bar{v},p\right\};\hat{v}\right) & ={\left(\bar{w},\rho \left\{\frac{\partial \bar{v}}{\partial t}+\left(\bar{v}-\hat{v}\right).\nabla \bar{v}\right\}\right)}_{\Omega_{t}}\\ & -{\left(\nabla .\bar{w},p\right)}_{\Omega_{t}}+{\left(\nabla^{s}\bar{w},2\mu \nabla^{s}\bar{v}\right)}_{\Omega_{t}}+{\left(q,\nabla .\bar{v}\right)}_{\Omega_{t}}\;,\text{and} \end{array}$$ (2b)

$$F_{G}\left(\bar{w}\right)={\left(\bar{w},\rho {\bar{f}}_{b}\right)}_{\Omega_{t}}+{\left(\bar{w},\bar{h}\right)}_{\Gamma_{t}^{N}}$$ (2c)

In Eq 2, quantities in the parentheses represent the inner product over the domain Ω_*t*_; *ρ* and *μ* are the fluid density and viscosity, respectively; $\bar{v}$ and *p* are the fluid velocity and pressure, respectively; $\hat{v}$ is velocity of the domain boundary or the endocardial wall obtained from image data; ${\bar{f}}_{b}$ is body force per unit volume; ∇^*s*^ is the symmetrization of the gradient operator ∇; $\left(S_{\bar{v}},S_{p}\right)$ and $\left(V_{\bar{v}},V_{p}\right)$ are the standard finite element solution and weighting function spaces defined on the computational domain, Ω_*t*_, respectively; $\Gamma_{t}^{D}$ and $\Gamma_{t}^{N}$ are the Dirichlet and the Neumann parts of the boundary, respectively, and $\bar{g}\left(\bar{x},t\right)$ and $\bar{h}\left(\bar{x},t\right)$ are the prescribed solution function on $\Gamma_{t}^{D}$ and the boundary traction vector on $\Gamma_{t}^{N}$, respectively.

As it is convenient to generate tetrahedral meshes for the complex ventricular cavities formed by the zebrafish endocardium, we desire to employ P1-P1 type (i.e., linear and continuous) spatial approximation of the fluid velocity and pressure solution variables. However, it is well known that the above Galerkin form of the Navier-Stokes equations (Eq 2) does not meet the Ladyzhenskaya-Babus̆ka-Brezzi (LBB) conditions (also known as *inf-sup* conditions) when discretized in space with equal-order interpolation functions, leading to an unstable scheme. [54, 55] Therefore, we use the variational multiscale (VMS) method which shares aspects of the pressure stabilizing/Petrov-Galerkin (PSPG) stabilization that circumvent LBB conditions, [53] and also shares aspects from the streamline upwind/Petrov-Galerkin (SUPG) stabilization to address the convective instability associated with the traditional Galerkin method. [40, 56]

Thus, the system of equations in the VMS formulation may be obtained as,

$$\begin{array}{c} B_{MS}\left(\left\{{\bar{w}}^{h},q^{h}\right\};\left\{{\bar{v}}^{h},p^{h}\right\};{\hat{v}}^{h}\right)-F_{MS}\left({\bar{w}}^{h}\right)=0\;\;,\;\text{where}, \end{array}$$ (3a)

$$\begin{array}{ll} B_{MS}(\{{\bar{w}}^{h},q^{h}\};\{{\bar{v}}^{h},p^{h}\};{\hat{v}}^{h}) & =B_{G}(\{{\bar{w}}^{h},q^{h}\};\{{\bar{v}}^{h},p^{h}\};{\hat{v}}^{h})\\ & +{\left(\left({\bar{v}}^{h}-{\hat{v}}^{h}\right).\nabla {\bar{w}}^{h},\tau_{M}{\bar{r}}_{M}\right)}_{\Omega_{t}}-{\left({\bar{w}}^{h},\tau_{M}{\bar{r}}_{M}.\nabla {\bar{v}}^{h}\right)}_{\Omega_{t}}\\ & -{\left(\nabla {\bar{w}}^{h},\frac{1}{\rho }\tau_{M}{\bar{r}}_{M}⊗\tau_{M}{\bar{r}}_{M}\right)}_{\Omega_{t}}\\ & +{\left(\nabla q^{h},\frac{1}{\rho }\tau_{M}{\bar{r}}_{M}\right)}_{\Omega_{t}}+{\left(\nabla .{\bar{w}}^{h},\rho \tau_{C}r_{C}\right)}_{\Omega_{t}}\\ & +{\left(\tau_{M}{\bar{r}}_{M}.\nabla {\bar{w}}^{h}\;\tau_{B},\tau_{M}{\bar{r}}_{M}.\nabla {\bar{v}}^{h}\right)}_{\Omega_{t}}\;\;,\text{and} \end{array}$$ (3b)

$$F_{MS}\left({\bar{w}}^{h}\right)=F_{G}\left({\bar{w}}^{h}\right),$$ (3c)

where *B*_*G*_ and *F*_*G*_ are defined in Eq (2), and the following definitions are used in Eq (3) where *n*_*sd*_ is the number of spatial dimensions

$${\bar{r}}_{M}=\rho (\frac{\partial {\bar{v}}^{h}}{\partial t}+({\bar{v}}^{h}-{\hat{v}}^{h}).\nabla {\bar{v}}^{h})+\nabla p^{h}-\mu \nabla^{2}{\bar{v}}^{h}-\rho {\bar{f}}_{b}^{h}$$ (4a)

$$r_{C}=\nabla .{\bar{v}}^{h}$$ (4b)

$$\tau_{M}={\left(\frac{C_{t}}{\Delta t^{2}}+\left(\bar{v}-\hat{v}\right).\bar{\bar{G}}\left(\bar{v}-\hat{v}\right)+C_{I}{\left(\frac{\mu }{\rho }\right)}^{2}\bar{\bar{G}}:\bar{\bar{G}}\right)}^{-1/2}$$ (4c)

$$\tau_{C}={\left(\tau_{M}\bar{g}.\bar{g}\right)}^{-1}$$ (4d)

$$\tau_{B}={\left(\tau_{M}{\bar{r}}_{M}.\bar{\bar{G}}\tau_{M}{\bar{r}}_{M}\right)}^{-1/2}$$ (4e)

$$G_{ij}=\sum_{k=1}^{n_{sd}}\frac{\partial \xi_{k}}{\partial x_{i}}\frac{\partial \xi_{k}}{\partial x_{j}}$$ (4f)

$$g_{i}=\sum_{j=1}^{n_{sd}}\frac{\partial \xi_{j}}{\partial x_{i}}\;.$$ (4g)

We employ the second-order generalized-*α* method for time integration, [57] linear finite elements (P1-P1) for spatial discretization, and a modified Newton-Raphson method for the linearization of the nonlinear terms in Eq 3. [39, 40] Additionally, we also solve for the mesh motion using linear elastostatics augmented by Jacobian-based stiffening. [58] We use a block iterative approach (called *quasi-direct* coupling) to solve the fluid-mesh system (Eq 3) in which the mesh motion lags behind the fluid system by one iteration. We employ the generalized minimal residual (GMRES) method with Jacobi preconditioning for solving the sparse system of linear equations. [40, 59] The solver is parallelized using the message passing interface (MPI) and has been optimized for performance on large scale computing clusters with efficient data management, cardiovascular blood flow modeling, and fluid-structure interaction (FSI). [19, 39, 60–63] We also employ a backflow stabilization method that is stable, yet minimally intrusive to avoid unphysiological flow reversal at the boundaries with Neumann boundary conditions. [19]

A common issue with simulating fluid dynamics in large deformation moving domains with conforming meshes is that the initial mesh quality deteriorates with large deformations and can create self-intersecting elements. As a result, one has to perform remeshing followed by data interpolation of the solution variables from the old mesh to the new mesh to advance the flow simulation. In the present study, we couple our flow solver with the TetGen open-source meshing library [64] to perform dynamic remeshing ‘on the fly’ to maintain the mesh quality. The element Jacobian (*J*_*e*_) is used as the metric to determine the mesh quality and remeshing is triggered when *J*_*e*_ ≤ 0. After remeshing, we perform data interpolation using an octree-based grid-to-grid advancing front vicinity search algorithm, [65] implemented in an MPI-based parallel environment. To simulate ventricular blood flow in zebrafish embryos, we begin with a volumetric tetrahedral mesh of ∼3 million elements at the end-systolic phase with edge size Δ*x* = 1.2*μm*. Due to dynamic remeshing, we create about 7-10 million elements in the ventricle by end-diastole, the absolute number varying slightly with the cardiac anatomy and function.

We previously performed validation of our flow solver to compare the simulated velocity field against 2-D PIV data in zebrafish embryos. In Lee et al., [32] we obtained reasonable agreement between PIV-based velocity acquisition and simulation predictions at the atrio-ventricular canal of the zebrafish embryos. Additionally, using the same solver, we also performed benchmark fluid dynamics and FSI tests for code verification in Esmaily-Moghadam et al. [39] and against experimental data for cardiovascular applications. [66, 67] We also validated our solver with dynamic remeshing against PIV-based three-dimensional three-component (3D-3C) velocity measurements of ventricular hemodynamics and demonstrated a reasonable agreement with measured data. [68, 69]

For boundary conditions we assume that the valves are fully developed at 4dpf, which agrees with observation from the image data (see S2 Movie in the Supplementary Material) and with previous studies. [18, 70, 71] During diastole, we apply a traction-free (Neumann) condition on the inflow boundary that mimics a fully-opened valve with a backflow stabilizing coefficient *β* = 0.3, and a homogeneous Dirichlet boundary condition on the outflow boundary to simulate total valve closure. Likewise, during systole we apply a traction-free condition on the outflow boundary and a homogeneous Dirichlet boundary condition on the inflow boundary. Assuming that the valves are fully formed, a positive value of the rate of change of volume of the ventricle indicates filling phase or diastole, and a negative value of the rate of change of ventricular volume implies ejection phase or systole, and the transition between these two phases occurs when the rate of change of ventricular volume is zero.

The endocardial wall velocity is computed from the motion detected during registration and is prescribed on the moving wall as a Dirichlet boundary condition. A linear interpolation of the velocity is performed at the intermediate flow time steps where the image data and the corresponding endocardial velocity is not available. Simulations are run with a time step of Δ*t* = 0.2*ms* on XSEDE resources (Comet supercomputing cluster, [72]) utilizing about 240 computing cores. The Courant–Friedrichs–Lewy number $\text{CFL}\equiv \frac{U_{p}\Delta t}{\Delta x}$, associated with numerical stability is about 2, and the flow Reynolds number $\text{Re}\equiv \frac{\rho U_{p}D_{i}}{\mu }$ is about 20 for wild type fish, based on peak average flow velocity (*U*_*p*_) and the diameter of the inflow annulus (*D*_*i*_). Each flow simulation requires about 1.5 days per cardiac cycle and the cost of remeshing and interpolation is ∼2% of the total simulation time. We simulate 4 cardiac cycles for each fish and compute the phase average of the last 3 cardiac cycles ignoring the initial transient effects for subsequent hemodynamic analysis.

#### Cardiac contractility indices

We define two measures of cardiac contractile function, stroke volume (SV) and ejection fraction (EF). The maximum volume of the ventricular endocardium during the cardiac cycle is the end-diastolic volume (EDV) and its minimum volume during the cardiac cycle is the end-systolic volume (ESV). Cardiac stroke volume (SV) is defined as the net volume of blood pumped by the ventricle in a single beat, quantified by the difference between EDV and ESV. The ratio of SV to EDV expressed as a percentage is known as the ejection fraction (EF). For adult healthy human hearts, left ventricular EF is typically 55 − 60%.

#### Shear stress and viscous dissipation

Wall shear stress (WSS) is thought to be intimately linked with cardiac trabeculation and morphogenesis. [4, 33] WSS has also been shown to influence endothelial cell alignment and direction, as well as the vascular growth and remodeling and aneurysm formation in prior studies. [73, 74] WSS (*τ*_*w*_) can be computed from the velocity field provided by the CFD simulation by taking the tangential component of the stress vector as,

$$\begin{array}{cc} & {\bar{\tau }}_{n}=2\mu (\nabla^{s}\bar{v})\bar{n}\\ & \text{WSS}\equiv {\bar{\tau }}_{w}\left(\bar{x},t\right)={\bar{\tau }}_{n}-({\bar{\tau }}_{n}.\bar{n})\bar{n}, \end{array}$$ (5)

where $\bar{n}$ is the endocardial surface normal vector. For a quantitative comparison of the shear profiles between the zebrafish variants, we compute area-averaged WSS (AAWSS) and space-time averaged WSS (AWSS) which is averaged in both space and time during the cardiac cycle, defined as,

$$\text{AAWSS}(t)=\frac{1}{A_{e}}\int_{\Gamma_{t}}|{\bar{\tau }}_{w}\left(\bar{x},t\right)|\;d\Gamma_{t}$$ (6a)

$$\text{AWSS}=\frac{1}{T_{c}A_{e}}\int_{t}^{t+T_{c}}\int_{\Gamma_{t}}|{\bar{\tau }}_{w}\left(\bar{x},t\right)|\;d\Gamma_{t}\;dt,$$ (6b)

where Γ_*t*_ is the endocardium surface with area *A*_*e*_, *T*_*c*_ is the cardiac cycle duration, and |(.)| represents the magnitude of the shear stress vector (${\bar{\tau }}_{w}$). To avoid high shear regions caused by entrance effects, we neglect the regions that were artificially extruded at the inlet and outlet annuli when computing the above integrals.

Like WSS, the oscillatory shear stress has been experimentally shown to influence the development of cardiac trabeculation. [75, 76] We compute non-dimensionalized oscillatory shear index (OSI) as,

$$\begin{array}{c} \text{OSI}=\frac{1}{2}\left(1-\frac{\left|\frac{1}{T_{c}}\int_{t}^{t+T_{c}}{\bar{\tau }}_{w}\;dt\right|}{\frac{1}{T_{c}}\int_{t}^{t+T_{c}}\left|{\bar{\tau }}_{w}\right|\;dt}\right), \end{array}$$ (7)

where the range of OSI varies between 0 and 0.5. An OSI value of 0 indicates that the shear stress vector is aligned along the same direction throughout the cardiac cycle, whereas an OSI of 0.5 indicates that the shear vector undergoes a 180° change in direction on a time-averaged basis and that the endothelial wall is subjected to highly oscillatory shear force.

Another fluid dynamical quantity of particular relevance to ventricular trabeculations is the viscous dissipation. [77] The presence of trabeculations can lead to enhanced energy dissipation that converts kinetic energy of the fluid into heat. Volume averaged kinetic energy density ($\bar{\text{KE}}$) and the rate of viscous dissipation ($\bar{\Phi }$) are mathematically expressed as,

$$\bar{\text{KE}}=\frac{1}{V_{d}}\int_{\Omega_{t}}\frac{1}{2}\rho {\left|\bar{v}\right|}^{2}\;d\Omega_{t}$$ (8)

$$\bar{\Phi }=\frac{1}{V_{d}}\int_{\Omega_{t}}\mu (\nabla^{s}\bar{v}\;:\nabla^{s}\bar{v})\;d\Omega_{t},$$ (9)

where *V*_*d*_ is the ventricular domain volume, *ρ* and *μ* are the density and viscosity of the fluid, respectively.

#### Zebrafish embryo treatments

Zebrafish embryos (*danio rerio*) are commonly used for cardiac developmental studies. Major factors attributed to this widespread usage are their small size, optical clarity, amenability to genetic manipulations and chemical treatment, rapid developmental period, and their ability to survive for several days without blood circulation but with sustained nutrition supply through passive diffusion. [78]

We use zebrafish embryos to develop insights into the mechanisms underlying the formation of cardiac trabeculation. We purchased transgenic (Tg(cmlc:gfp)) wild type zebrafish from UCLA zebrafish core lab facility and since the cardiac myosin light chain (cmlc) contains green fluorescent protein (gfp), we were able to image the zebrafish heart motion with 473*nm* wavelength laser without any additional preparation or treatment. We then performed genetic manipulation and chemical treatment on the wild type disrupting the normal cardiac trabeculation.

In particular, an *ErbB2* signaling inhibitor, AG1478, was used to inhibit differentiation and proliferation of trabecular myocytes during cardiac morphogenesis. The injection of *gata1a morpholino oligomer* (*gata1aMO*) at 1-4 cell stages of developing embryo inhibited red blood cell production (hematopoiesis), thereby reducing blood viscosity and endocardial WSS, resulting in attenuated trabeculation. Also, a *weak atrium*^*m*58^ (wea) mutant was used to inhibit atrial contraction, leading to reduced blood flow through ventricles during peristaltic contractions, resulting in a highly under-developed ventricle with poor cardiac function. We assume that the normal valvulogenesis is not affected due to chemical and genetic manipulations, justified by our observations of normal valve function in the image data at the time point we are simulating. [18, 70, 71] Therefore, our treatment of boundary conditions for wild type fish can be applied to the treated fish types as well.

**Fig 4:**
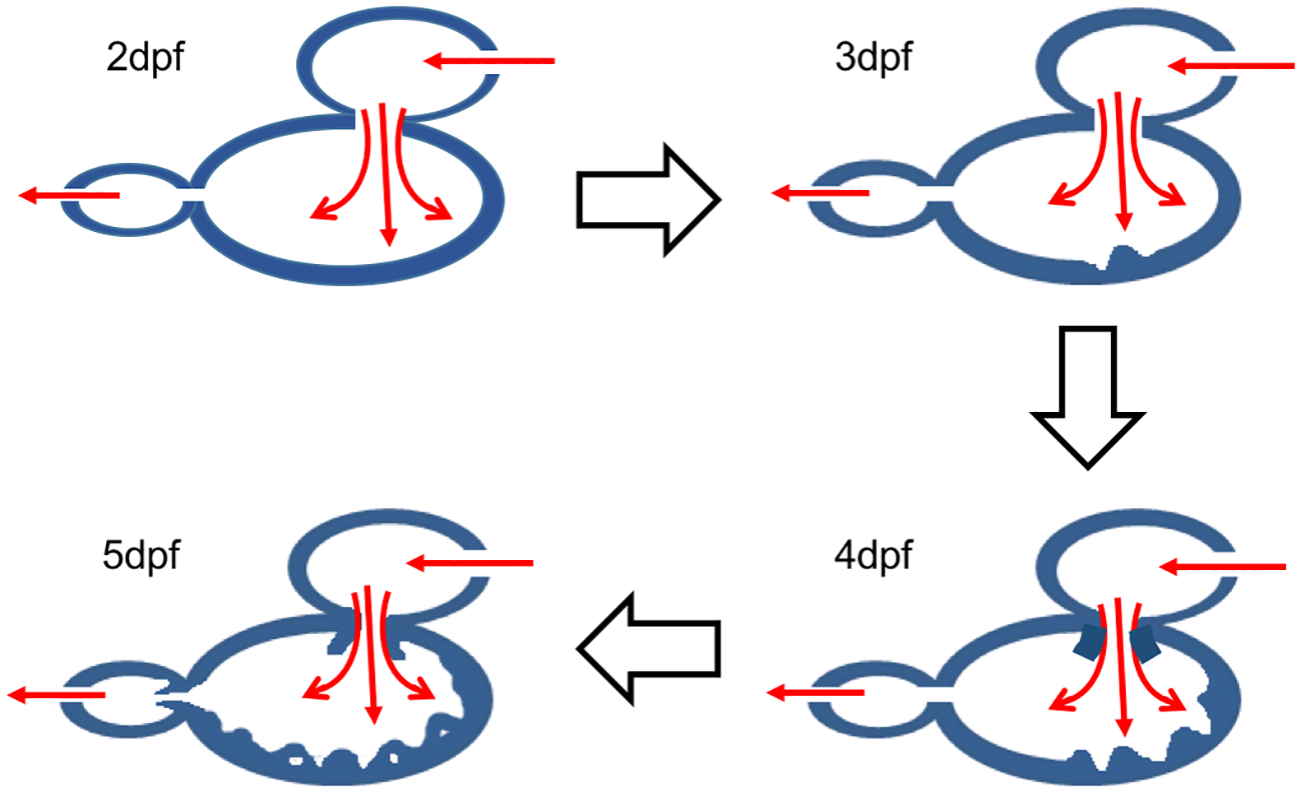
A schematic of the development of trabeculations in the wild type zebrafish. At 2dpf, we do not see any noticeable trabeculations in the ventricular myocardium. At 3dpf and 4dpf, the trabeculations are developed in the form of ridges and grooves. At 5dpf the trabeculations further developed into sponge-like network. [9] Colored arrows indicate direction of the blood flow whereas block arrows indicate progress of the developmental stages.

Next, we performed 4-D light-sheet SPIM imaging [33] to capture the contracting hearts at 4 days post fertilization (dpf) for all the fish types. While the wild type fish developed cardiac trabeculation in the form of ridges and grooves, (Fig 4) the trabeculation was attenuated in the treated zebrafish ventricles. We demonstrated a nearly 42% reduction in the volume of the trabeculations in fish treated with *gata1aMO* compared to the wild type by calculating the myocardium volume in the trabeculation grooves based on the SPIM images. [33] However, we do not find any visible trabeculations in the images of AG1478 and *wea-mutant*. For the wild type zebrafish and each treated variant described, we performed image registration to extract wall motion data, performed moving-domain blood flow simulations followed by post-processing to compute the above described hemodynamic quantities. We have used the same values of blood density (*ρ* = 1.06*g*/*cm*^3^) and viscosity (*μ* = 4*cP*) for all fish types to perform blood flow simulations. We also consider an additional case for *gata1aMO* where the viscosity is reduced as a result of inhibiting hematopoiesis. In Vermot et al. [18], it was demonstrated that introducing *gata1MO* reduces viscosity by 90% and treating with *gata2MO* reduces viscosity by 70%. Therefore, we chose an intermediate value of 75% as the viscosity reduction factor due to *gata1aMO* injection. We note that the baseline viscosity value employed in the present study is for an adult zebrafish (*μ* = 4*cP*, hematocrit (*Ht*) = 35%). [79]

## Results

### Cardiac function and contractility

**Fig 5:**
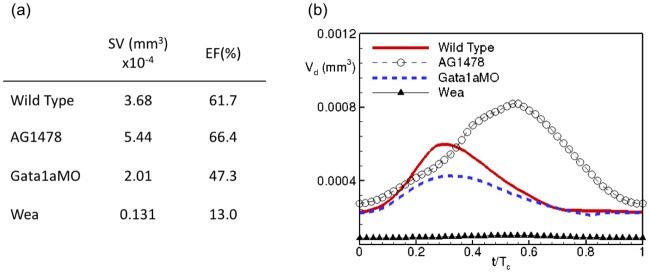
Comparison of (a) contractility indices, and (b) ventricular volume as a function of the non-dimensionalized cardiac cycle duration, between wild type zebrafish and in response to chemical (AG1478) and genetic (*gata1aMO*, *wea*) manipulations. SV: stroke volume, EF: ejection fraction, *V_d_*: ventricular cavity volume, *T_c_*: cardiac cycle duration.

We observe a substantial change in cardiac contractility due to chemical treatment with AG1478 compared with the *gata1aMO* injected fish and the *wea* mutant (Fig 5). In response to chemical treatment AG1478, SV increases by 48% whereas EF marginally increases by ∼7% compared to the wild type (Fig 5a). Genetically treated *gata1aMO* and *wea* have reduced cardiac contractility with respect to the wild type. For *gata1aMO*, SV is smaller by 45% and EF is smaller by 23% compared to the wild type (Fig 5a). On the other hand, *wea* mutant has a significantly smaller SV and EF (Fig 5a).

We note similar trends in the ventricular volume variation during the cardiac cycle for all the fish types (Fig 5b). Additionally, we note a prolonged diastole (ventricular filling) in response to AG1478 treatment, such that the ventricle reaches a maximum volume much later in the cardiac cycle (Fig 5b). On the other hand, the ventricular volume variation for *gata1aMO* and *wea* remains nearly in phase with the wild type (Fig 5b).

### Hemodynamic shear

**Fig 6:**
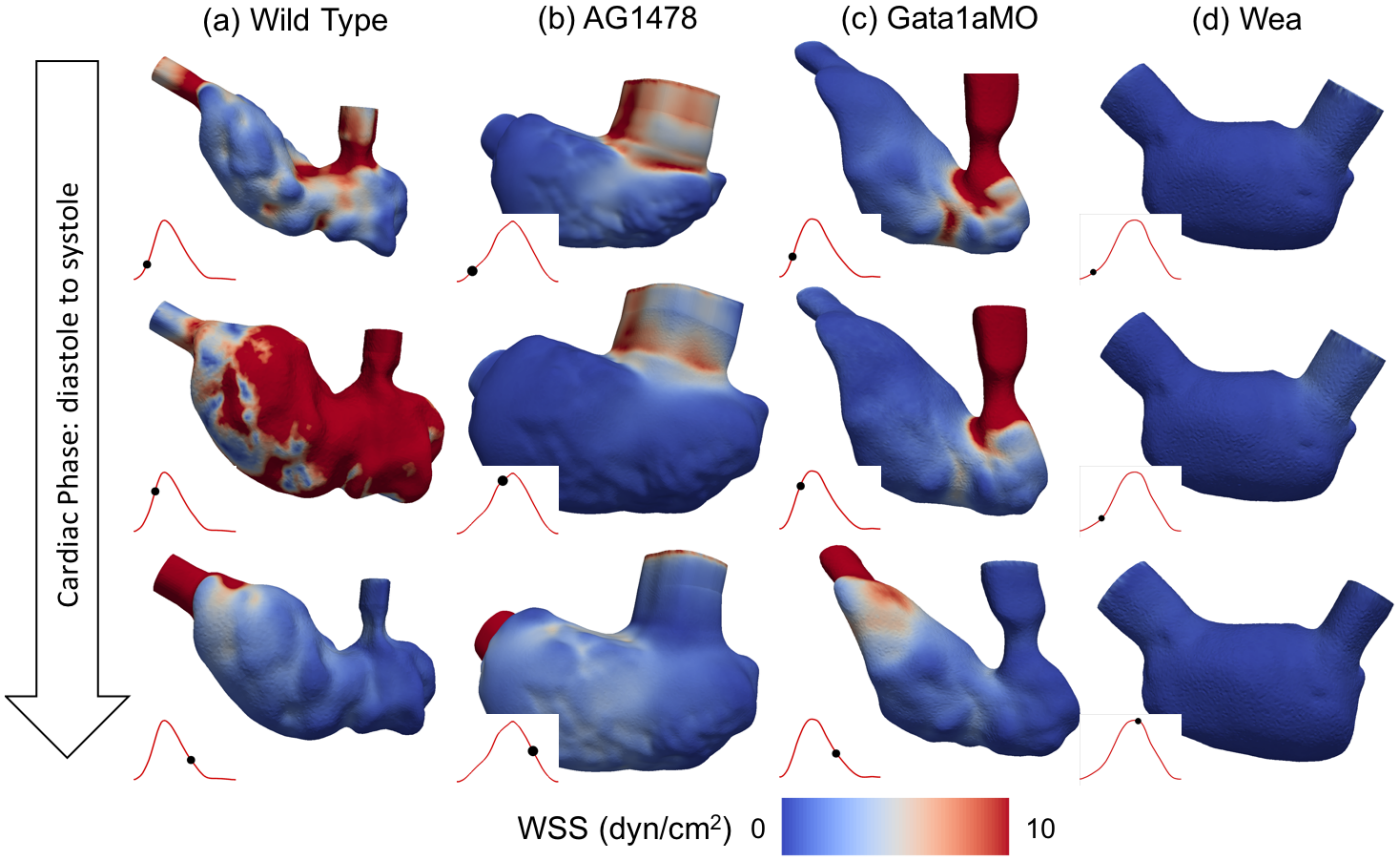
Endocardial wall shear stress (WSS) profiles are compared at different cardiac phases (rows) corresponding to early diastole, mid-diastole and mid-systole, between the wild type zebrafish embryos and in response to chemical and genetic treatments (a) wild type, (b) AG1478, (c) *gata1aMO*, (d) *wea*. The red line in each figure represents ventricular volume variation and the black dot identifies the corresponding instant during the cardiac cycle. All the phases are chosen to be at the same non-dimensionalized time with respect to the cardiac cycle duration (*T_c_*) of each fish. This figure also illustrates the differences in ventricular morphology (volume and deformation) during the cardiac cycle for the chemically and genetically altered fish.

Localized zones of high and low WSS occur in the wild type zebrafish at early diastole (top frame of Fig 6a), which correspond to the sites of the trabecular ridges and grooves, respectively. On the other hand, AG1478 and *gata1aMO* have more uniformly low WSS on the ventricular surface, except for the sites of inflow jet impingement. The regions of higher WSS occurring in the artificial inflow and outflow annuli extensions are not included in our analysis. Further into the cardiac cycle (middle row, Fig 6), WSS is higher over most of the ventricular endocardium for the wild type zebrafish, whereas the WSS is lower for both AG1478 and *gata1aMO*. During systole (bottom row, Fig 6), mild variation in WSS is noted around the sites of trabeculations in the wild type model, which are not present in AG1478 and *gata1aMO* treated fish. In the case of *wea* mutant (Fig 6d), WSS is uniformly low throughout the cardiac cycle, which is attributed to its poor cardiac contractility and function (Fig 5).

**Fig 7:**
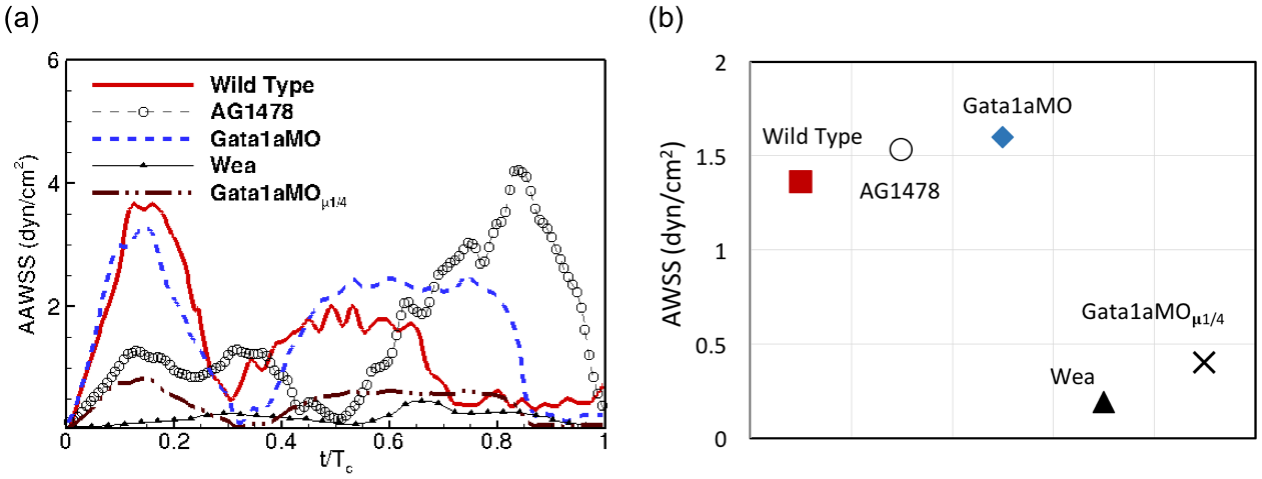
Comparison of (a) area-averaged WSS (AAWSS) as a function of non-dimensional time, (b) time-averaged wall shear stress (AWSS), between the wild type zebrafish and in response to chemical (AG1478) and genetic (*gata1aMO*, *wea*) treatments. We also include an additional case for genetically treated *gata1aMO* with blood viscosity reduced by a factor of 4 (*gata1aMO*_*μ*1/4_) to account for inhibited hematopoiesis.

In Fig 7a, we note that the diastolic and systolic behavior of AAWSS appear to be reversed between the wild type and genetically manipulated fish types (*gata1aMO, wea*), and the chemically treated AG1478. First, we observe a diastolic peak of AAWSS for the former group, whereas the latter has a systolic peak. Second, during diastole, AAWSS rises and falls more sharply for the wild type and the genetic variants (*gata1aMO, wea*), whereas it plateaus for the chemically treated AG1478. However, during systole the trend is reversed between the two groups.

The time-averaged shear stress (AWSS) differs slightly between the wild type and AG1478 (Fig 7b). On the other hand, *gata1aMO* exhibits a higher time-averaged value compared to the wild type. This is due to a higher systolic shear that is spread over a wider range of the cardiac cycle for *gata1aMO* compared to the wild type (Fig 7a). Nevertheless, with a reduced viscosity (*gata*1*aMO*_*μ*1/4_), the AWSS of *gata1aMO* is significantly lower compared to both the wild type and AG1478 (Fig 7b). The shear profile for *wea* mutant is consistently low over the cardiac cycle (Fig 7a), as is the time average (Fig 7b).

**Fig 8:**
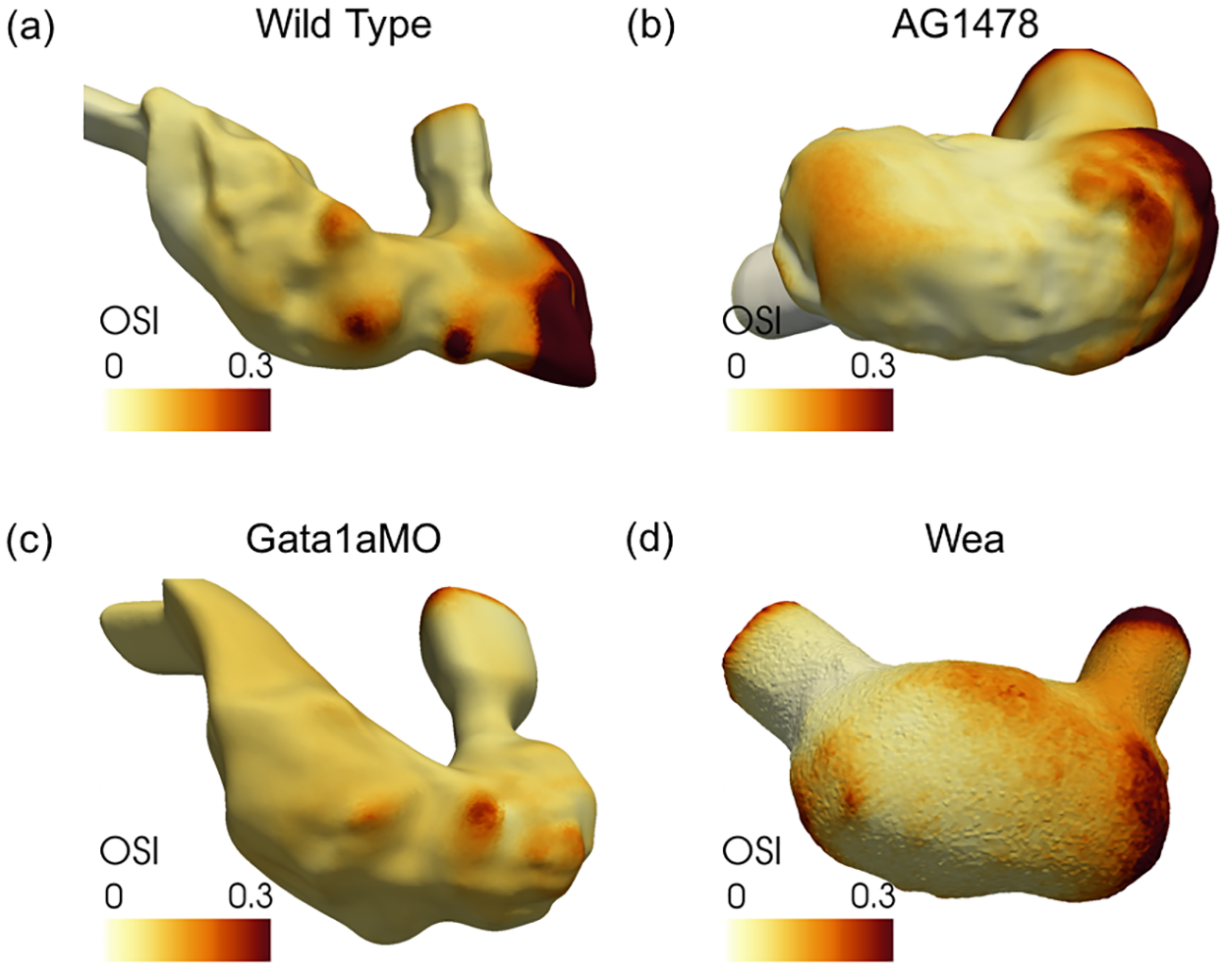
Oscillatory shear index (OSI) comparison of OSI profiles on the ventricular surface between wild type and the chemically and genetically treated zebrafish. OSI is higher in the trabecular grooves but lower in the trabecular ridges and on the rest of the smooth endocardium for the wild type. On the other hand, we observe fewer sites with high OSI for the treated fish types (AG1478, *gata1aMO* and *wea*).

In Fig 8a for the wild type, OSI is higher in the trabecular grooves, but lower in the trabecular ridges and on the rest of the smooth endocardium. On the other hand, we observe fewer sites with high OSI for the treated fish types (AG1478, *gata1aMO* and *wea*).

### Energy dissipation

**Fig 9:**
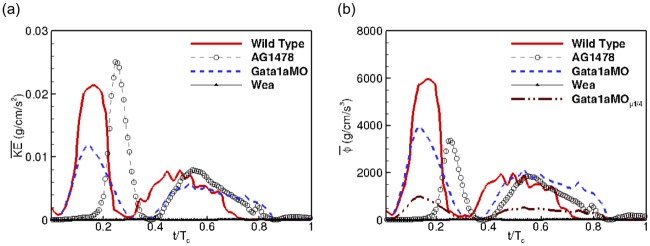
Comparison of (a) kinetic energy density ($\bar{\text{KE}}$), and (b) the rate of viscous dissipation per unit volume ($\bar{\Phi }$) during the cardiac cycle, between the wild type zebrafish and in response to chemical (AG1478) and genetic (*gata1aMO*, *wea*) treatments.

Both $\bar{\text{KE}}$ and $\bar{\Phi }$ exhibit a two-peak profile during the cardiac cycle (Fig 9). While the first peak occurs during early diastole and varies sharply with a narrow spread, the second peak occurs between mid-diastole and mid-systole with a wider spread and a lower peak value. We also note that the shapes of the time variation of the kinetic energy density $\bar{\text{KE}}$ and the rate of viscous dissipation $\bar{\Phi }$ curves are similar for all fish types but only differ in magnitude (Fig 9).

We note that AG1478 has higher peak value of kinetic energy $\bar{\text{KE}}$ (Fig 9a) but substantially reduced dissipation $\bar{\Phi }$ (Fig 9b) compared to the wild type. The genetically treated *gata1aMO* has a lower peak $\bar{\text{KE}}$ and $\bar{\Phi }$ compared to the wild type. However, this difference in energy budget between *gata1aMO* and the wild type is reduced during late diastole to early systole, and by late systole, the *gata1aMO* has marginally higher kinetic energy and dissipation compared to the wild type (Fig 9). Lowering the blood viscosity by 4 (*gata1aMO_1/4_*) results in reduced energy dissipation compared to either wild type or AG1478 (Fig 9b). Both $\bar{\text{KE}}$ and $\bar{\Phi }$ are an order of magnitude lower for *wea* mutant fish and are therefore negligible (Fig 9).

## Discussion

It is well known that fluid forces such as hemodynamic shear and pressure gradients orchestrate biological responses that affect downstream embryonic heart development. [1, 4] However, quantifying these forces in developing embryos *in vivo* or *in vitro* is a significant challenge. [3, 4] Computational modeling offers a promising methodology to quantify these biomechanical forces, providing space-time resolved blood flow data in developing hearts. However, simulating hemodynamics in moving heart chambers requires specialized numerics to account for the moving wall. Acquiring high-resolution images of beating hearts in developing embryos and extracting the deforming ventricular geometry from these images for flow simulations, presents further challenges which had not been adequately addressed in prior studies.

We address these challenges in the present study with a novel computational framework combining 4D-light sheet imaging and a validated moving-domain blood flow solver for measuring ventricular hemodynamic forces in developing zebrafish hearts. While we have demonstrated the utility of the framework for zebrafish embryos, the framework could be generalized to other developmental studies in chicks and mice with appropriate time-resolved imaging modalities. We have previously demonstrated the 4-D imaging methodology using light sheet microscopy coupled with a non-gated synchronization algorithm, and applied it to investigate Notch signaling mechanisms during cardiac morphogenesis. [33] In the current study, we couple the 4-D light sheet imaging with the blood flow solver to quantify the biomechanical forces, which can then be used to elucidate hemodynamic regulatory mechanisms during cardiac development.

4-D light sheet microscopy provides high spatio-temporal resolution images of beating hearts of developing embryos with high heart rates and large endocardial deformations. In the case of zebrafish, the technique offers the added advantage that the transparent embryos do not have to be sacrificed at the end of the experiment, and this allows one to track temporal changes during development. However, the high spatio-temporal resolution of these images poses a challenge in extracting the motion of the beating heart because segmenting the endocardium at each time frame is a labor-intensive and error-prone process. In the present framework, we employ robust and efficient deformable image registration methods allowing the user to segment only one cardiac phase and extract the motion of the ventricle from the displacement field computed during registration. Hence, the 4-D motion of the deforming ventricle can be extracted in a small fraction of the time required for blood flow simulations. Our framework employs stabilized multiscale finite element methods based on the ALE formulation to solve blood flow in moving domains, which has been well-established and validated application in cardiovascular disease and developmental cardiology. [21, 32]

We have applied the framework in wild type and three treated variants of zebrafish embryos to examine the mechanobiology during cardiac trabeculation. We categorize these treatments under direct and indirect approaches to attenuating cardiac trabeculation in developing zebrafish embryos. Chemical treatment with AG1478 directly knocks out a key component of endocardial Notch signaling pathway responsible for differentiation and proliferation of trabecular myocytes. However, *gata1aMO* injection is an indirect method of attenuating trabeculation by passively reducing hemodynamic shear on the ventricular endocardium. This reduction in shear is a consequence of inhibiting red blood cell production (hematopoiesis) leading to low blood viscosity. The *wea* mutant also indirectly knocks out trabeculation due to inhibited peristaltic contractions of atrium resulting in an underdeveloped ventricle. We have compared multiple quantities among these fish variants, including the cardiac contractile function, hemodynamic shear related metrics, kinetic energy and viscous dissipation, and related these dynamic quantities with the development of ventricular trabeculations.

We showed that chemically treating the zebrafish embryo with AG1478 leads to an untrabeculated ventricle with a significant increase in stroke volume (↑48%) but a marginal 7% increase in ejection fraction. We also showed that AG1478 treatment leads to prolonged diastole compared to other fish variants. However, we note that zebrafish are known to have significant variability across the species, and it is not immediately clear how much of this prolongation is attributed to AG1478 treatment alone. Therefore, we can postulate that AG1478 treatment leads to an untrabeculated dilated ventricle but nearly preserves the cardiac contractile function. However, the genetically manipulated varieties *gata1aMO* and *wea*, not only attenuated cardiac trabeculation but also led to reduced cardiac function and contractility. In particular, the case of *wea* mutant exhibits an order of magnitude reduction in ventricular size and SV, and significantly reduced EF. These observations are consistent with our experience with multiple fish subjected to genetic and chemical alterations.

We have demonstrated high and low oscillatory shear juxtaposed in the trabecular grooves and ridges, respectively for the wild type zebrafish. We have also demonstrated spatial inhomogeneity of WSS for the wild type fish. However, none of the treated variants exhibit the same spatial variations in endocardial WSS, and all have lower OSI uniformly distributed on the endocardium. On the other hand, chemical treatment in AG1478 and *gata1aMO* injection resulted in similar averaged shear compared to the wild type. Altering the viscosity of the blood due to *gata1aMO* injection, however, resulted in lowering the average shear. This suggests that average shear may not be a strong factor responsible for regulating cardiac trabeculation; but spatial inhomogeneity of endocardial WSS and oscillatory shear forces are implicated in the development of cardiac trabeculation.

Comparison of the energy budget between the fish variants shows that kinetic energy density correlates with cardiac function. Compared to the wild type zebrafish, AG1478 has marginally higher $\bar{\text{KE}}$ while *gata1aMO* and *wea* mutant fish have substantially reduced $\bar{\text{KE}}$. On the other hand, viscous dissipation ($\bar{\Phi }$) is enhanced by the presence of trabeculations, which act as surface roughness on the endocardial wall, leading to increased viscous losses. This phenomenon is evident in Fig 9b where dissipation is reduced for all the treated fish types compared to the wild type. With pronounced trabeculations in *gata1aMO* compared to AG1478, dissipation is marginally higher, despite having lower contractility and $\bar{\text{KE}}$. However, this marginally increased dissipation is only reflective of increased strain rates in the presence of trabeculations for *gata1aMO* and does not take into account the changes in viscosity from reduced hematopoiesis. Nevertheless, the changes in $\bar{\text{KE}}$ and $\bar{\Phi }$ amount to a negligible fraction of the total cardiac work, calculated from the area under the ventricular pressure-volume loop, corroborating similar recent claims for human cardiac physiology. [77] Typically, peak systolic and end-diastolic pressures in zebrafish embryos are 0.47 ± 0.09mmHg and 0.08 ± 0.07mmHg, respectively at 5dpf. [80] Thus, the cardiac work per unit volume of the zebrafish embryo at 5dpf is approximately 520g/cm/s^2^, and is therefore orders of magnitude higher than the maximum $\bar{\text{KE}}$ for wild type fish.

Our novel computational modeling framework can provide spatio-temporally resolved blood flow information, from which we extract biomechanical forces acting on the ventricular endocardium in the developing heart; these data are not yet possible to obtain by direct experimental measurements. The availability of such a framework fosters future opportunities to perform quantitative assessment of mechanobiological processes (for transduction and gene expression) in both health and disease. Spatio-temporal correlations of hemodynamic forces with gene expression and cellular signaling are enabled by this platform, which is currently not possible *in vivo* or with other limited experimental techniques such as PIV. Insights developed in these fundamental studies will pave the way for improved understanding of the role of embryonic hemodynamics on the onset of congenital heart disease.

We acknowledge several limitations in our study which could be addressed in future investigations.

First, we made a Newtonian flow assumption for modeling blood dynamics in the zebrafish embryo, although it is known that blood may exhibit non-Newtonian behavior at embryonic microscales. While many prior developmental studies have also modeled blood flow as Newtonian, [14, 30, 31] there is insufficient data on the non-Newtonian effects of blood in developing ventricles. Previous studies on the non-Newtonian dynamics of blood in human models of arteries demonstrated a nearly two-fold increase in WSS compared to the baseline Newtonian flow. [81] However, comparable shear profiles could also be obtained by rescaling the value of the viscosity used in the Newtonian flow model. [81] Conversely, Boyd et al. [82] demonstrated very small differences in both velocity profiles and shear rates assuming blood to be non-Newtonian, especially near the vessel wall, using a model of an ideal artery.

Our own recent capillary-pressure driven microchannel experiments indicated that the viscosity of blood is nearly independent of shear rates (Newtonian flow) when the shear rate is >500*s*^−1^, but that viscosity increases at lower shear rates (<500*s*^−1^) where non-Newtonian dynamics may become important. [79] By this metric, about 70% of the cardiac cycle is predominantly Newtonian with high shear rates (*O*(1000*s*^−1^)) in fish types with high ventricular contractility (wild type, AG1478 and *gata1aMO*). The shear rates are so low in the rest of the cardiac cycle that changes in viscosity due to non-Newtonian effects may be too small to substantially influence the WSS magnitude. In the case of *wea-mutant* with poor contractility, we do find very low shear rates with values of WSS orders of magnitude lower compared to other fish types. However, we postulate that modeling the non-Newtonian blood dynamics in the present study would not affect the relative WSS distributions and our major conclusions. Nevertheless, we also note that non-Newtonian phenomena could be implemented in our computational framework without disrupting the underlying workflow and should be a subject of future study.

Second, although we demonstrated the applicability of our framework in only one fish of each type, this is intended as a stepping stone to future investigations which statistically correlate gene expression data with hemodynamic forces.

Third, while we quantified differences in shear characteristics in response to chemical and genetic treatments compared to the wild type fish, these were demonstrated for the fish imaged at 4dpf. To understand how hemodynamic shear regulates the onset of cardiac trabeculations, future studies should examine the changes in endocardial shear characteristics at different stages post fertilization and correlate them with the ventricular morphology during the course of embryonic heart development. Additionally, other biomechanical forces such as pressure and its gradients, wall strains, etc. could also affect the mechanobiology during cardiac morphogenesis and should be examined in future investigations. [30, 32] Further, as our framework relies on imposed wall motion from 4D light sheet SPIM images and models blood flow as incompressible, we cannot determine wall strains and absolute blood pressure but only their gradients.

Fourth, although we have validated our flow solver against 2-D PIV measurements in developing zebrafish embryos, [32] and in other benchmark fluid dynamics tests, [21, 39] and also verified the image registration methodology by comparing the ventricular cavity volumes between manual segmentations and the ones obtained from image-based registration techniques, a more concrete validation study with 4-D *in vivo* measurements in developing embryos is warranted contingent upon the future availability of such data.

Lastly, the zebrafish treatments considered here were focused on inhibiting cardiac trabeculation; however, a possible future direction could be to examine the role of endocardial Notch signaling and shear forces on hypertrabeculation. Recent reports suggest that late DAPT treatment of embryonic zebrafish can lead to increased trabeculation, and we plan to pursue this direction in future work. [83]

### Conclusion

We have developed a novel computational framework to quantify time-dependent hemodynamic forces in developing embryos based on 4-D light sheet imaging data. Our efficient and streamlined workflow employs deformable image registration methods for extracting the endocardial motion, and is coupled with a stabilized variational multiscale finite element flow solver that is validated and optimized for modeling cardiac hemodynamics. We have demonstrated the workflow in wild type zebrafish and in three treated fish types disrupting normal cardiac trabeculation. These variants include: (a) chemical treatment using AG1478 that inhibits *ErbB2* signaling; (b) injection of *gata1a morpholino oligomer* (*gata1aMO*) suppressing hematopoiesis and thereby, reducing blood viscosity and shear; and (c) *weak-atrium*^*m*58^ mutant (*wea*) with attenuated atrial contraction. Our simulations revealed high oscillatory shear index (OSI) in the grooves between trabeculae compared to the ridges in wild type zebrafish, suggesting oscillatory forces to be implicated in cardiac trabeculation. Our analysis also indicates that the presence of endocardial trabeculations significantly enhances viscous losses in the wild type zebrafish compared to the treated variants, although, the magnitude of this increase is small compared to the total cardiac work. This framework is broadly applicable in other cardiac developmental studies focused on quantifying mechanobiologically relevant forces during morphogenesis.

## Supporting information

S1 Movie Registration. Motion of the registered zebrafish ventricle shows good adherence with the background image in multiple views.
(AVI)

S2 Movie Valve leaflets. Imaging transgenic (Tg(cmlc:mcherry;flk:gfp)) wild type zebrafish shows fully developed valve leaflets at 4dpf.
(RAR)
