# Correction: A method to quantify mechanobiologic forces during zebrafish cardiac development using 4-D light sheet imaging and computational modeling

**DOI:** 10.1371/journal.pcbi.1006482

**Published:** 2018-09-17

**Authors:** Vijay Vedula, Juhyun Lee, Hao Xu, C.-C. Jay Kuo, Tzung K. Hsiai, Alison L. Marsden

The Data Availability statement for this paper is incorrect. The correct statement is: All image segmentation, cardiac model construction and finite element software is available as part of the SimVascular open source project at www.simvascular.org. The registration toolkit is available at https://sites.google.com/site/myronenko/research/mirt. Image data and simulation results has been incorporated at the open repository http://simvascular.stanford.edu/downloads/public/open_data/zebrafish_PLOSCompBio_2017/
